# Upregulation of KLK8 Predicts Poor Prognosis in Pancreatic Cancer

**DOI:** 10.3389/fonc.2021.624837

**Published:** 2021-07-30

**Authors:** Qing Hua, Tianjiao Li, Yixuan Liu, Xuefang Shen, Xiaoyan Zhu, Pingbo Xu

**Affiliations:** ^1^Department of Anesthesiology, Shanghai Cancer Centre, Fudan University, Shanghai, China; ^2^Department of Oncology, Shanghai Medical College, Fudan University, Shanghai, China; ^3^Department of Anesthesiology, Zhongshan Hospital, Fudan University, Shanghai, China; ^4^Department of Pancreatic Surgery, Shanghai Cancer Centre, Fudan University, Shanghai, China; ^5^Shanghai Pancreatic Cancer Institute, Fudan University Shanghai, Shanghai, China; ^6^Pancreatic Cancer Institute, Fudan University, Shanghai, China; ^7^Department of Clinical Laboratory, Shanghai Cancer Centre, Fudan University, Shanghai, China; ^8^Department of Physiology, Navy Medical University, Shanghai, China

**Keywords:** apoptosis, kallikrein-related peptidase 8, pancreatic ductal adenocarcinoma, PI3K-Akt-mTOR pathway, proliferation

## Abstract

Pancreatic ductal adenocarcinoma (PDAC) is a growing cause of cancer-related mortality worldwide. Kallikrein-related peptidase 8 (KLK8) has potential clinical values in many cancers. However, the clinicopathological significances of KLK8 in PDAC remain unknown. We explored the relationship of KLK8 to clinicopathological features of PDAC based on public databases. KLK8 expression was examined in human PDAC tissues. Cell proliferation and apoptosis were evaluated in KLK8-overexpressed human pancreatic cancer cell lines Mia-paca-2 and Panc-1. The related signaling pathways of KLK8 involved in pancreatic cancer progression were analyzed by gene set enrichment analysis (GSEA) and further verified in *in vitro* studies. We found that KLK8 was up-regulated in tumor tissues in the TCGA-PAAD cohort, and was an independent prognostic factor for both overall survival and disease-free survival of PDAC. KLK8 mRNA and protein expressions were increased in PDAC tissues compared with para-cancerous pancreas. KLK8 overexpression exerted pro-proliferation and anti-apoptotic functions in Mia-paca-2 and Panc-1 cells. GSEA analysis showed that KLK8 was positively associated with PI3K-Akt-mTOR and Notch pathways. KLK8-induced pro-proliferation and anti-apoptotic effects in Mia-paca-2 and Panc-1 cells were attenuated by inhibitors for PI3K, Akt, and mTOR, but not by inhibitor for Notch. Furthermore, overexpression of KLK8 in Mia-paca-2 and Panc-1 cells significantly increased epidermal growth factor (EGF) levels in the culture media. EGF receptor (EGFR) inhibitor could block KLK8-induced activation of PI3K/Akt/mTOR pathway and attenuate pro-proliferation and anti-apoptotic of KLK8 in Mia-paca-2 and Panc-1 cells. In conclusion, KLK8 overexpression exerts pro-proliferation and anti-apoptotic functions in pancreatic cancer cells *via* EGF signaling-dependent activation of PI3K/Akt/mTOR pathway. Upregulated KLK8 in PDAC predicts poor prognosis and may be a potential therapeutic target for PDAC.

## Background

Pancreatic cancer is one of the most leading causes of cancer death in both males and females because of its poor prognosis, with almost as many deaths (n = 432,000) as cases (n = 459,000) (1). According to 2020 cancer statistics, approximately 57,600 new cases of pancreatic cancer will be diagnosed, killing almost 47,050 people in the United States in 2020, making it the fourth leading cause of cancer-associated death (2). Despite the advanced therapeutic approaches, the 5-year relative survival rate of pancreatic cancer remains poor, estimated at 9% (3–5). To further improve the survival rates, it is critical to identify a more sensitive and effective biomarker associated with the tumorigenesis and progression for early detection, which will improve the prognosis for pancreatic cancer.

Tissue kallikrein-related peptidases (KLKs) are a group of serine proteases encoded by 15 highly conserved genes (*KLK1-KLK15*) arranged in a tandem cluster (~300 kb) on chromosome 19q13.3–13.4 (6–9). KLK8, an important member of the KLKs family, is a synaptic, plasticity-modulating extracellular serine protease and has been found in many tissues and biological fluids, involved in a variety of biological activities, for instance, epidermal proliferation and differentiation, terminal differentiation of keratinocytes and so on (10, 11). Abnormal KLK8 expression has been found in several malignancies, including ovarian, cervical, gland and lung cancers (12–15). Meanwhile, accumulating evidence support the clinical utility of KLK8 as a biomarker for cancer survival and prognosis. However, the expression pattern and role of KLK8 in pancreatic cancer remains unknown.

In this study, we explored the expression of KLK8 in the pancreatic cancer at both the mRNA and protein levels and investigated the correlation between KLK8 expression and prognosis of pancreatic cancer patients. We also investigated whether and how KLK8 affected the proliferation and apoptosis of pancreatic cancer cells. Our findings demonstrated that upregulation of KLK8 was related to a poor prognosis in pancreatic cancer. Overexpression of KLK8 might promote proliferation and inhibit apoptosis *via* epidermal growth factor (EGF) signaling-dependent activation of PI3K/Akt/mTOR pathway in pancreatic cancer cells.

## Methods

### Patients and Specimens

Thirty pancreatic cancer tissue samples and their matched para-cancerous pancreas from pancreatic cancer patients who underwent surgery in Fudan University Shanghai Cancer Center (Fudan Center) between June 2016 and April 2018 were obtained during operations. All diagnoses were confirmed by two pathologists. All specimens were acquired after written informed consent following procedures approved by the Ethics Committee of Fudan University Shanghai Cancer Center.

### The Cancer Genome Atlas Analysis and GSEA

The GEPIA database provided differential gene expression analysis of 31 kinds of cancers based on integrated analysis of the TCGA and GTEx databases. TCGA Pancreatic Cancer (PAAD) cohort consisted of 178 primary pancreatic cancer and 4 normal samples. And gene expression of 167 normal pancreatic tissue was also downloaded from GTEx (http://commonfund.nih.gov/GTEx/) to explore the differential expressed genes between tumors and normal tissues. All data on expression and clinical features were obtained from the USUC Xena Cancer Genomics Browser (https://xenabrowser.net/datapages/?dataset=TCGA). The differentially expressed genes with |log2foldchange|≥1 and P < 0.05 were selected based on 178 pancreatic cancer samples and 171 normal pancreas samples. X-tile program (www.tissuearray.org/rimmlab/) was used to determine the optimum cutoff value of KLK8 with minimum p value defined by Kaplan–Meier survival analysis and log-rank test. To elucidate the mechanisms behind KLK8 in pancreatic cancer, Gene set enrichment analysis (GSEA) was performed on the Broad Institute Platform, and statistical significance (false discovery rate, FDR) was set at 0.25. Hallmark gene set collection was used to find relative signaling pathways of KLK8 from control and KLK8 overexpression group according to the genes presenting the strongest enrichment scores.

### RNA Extraction and Real-Time Quantitative Polymerase Chain Reaction (RT-qPCR)

Total RNA from 30 random pairs of fresh pancreatic cancer tissue and adjacent normal mucosa were isolated using the Trizol reagent (TaKaRa, Japan) according to the manufacturer’s instruction. Primers for RT-qPCR were designed using Primer Express v2.0 software (Applied BioSystems). The primer sequences for KLK8 were as follows: forward 5’- AAG TGCACC GTC TCA GGC-3’ and reverse 5’- TCC TCA CAC TTC TTC TGG GG-3’. β-actin was used as an internal control, and the primer sequences were as follows: forward 5’-CTA CGT CGC CCT GGA CTT CGA GC -3’ and reverse 5’- GAT GGA GCC GCC GAT CCA CAC GG -3’. Real-time PCR was carried out using SYBR Green I (Applied BioSystems) and the relative expression was calculated using the 2 ^-ΔΔCT^ method and normalized to β-actin (human) as the internal control gene.

### Immunohistochemical (IHC) Staining

The immunohistochemistry (IHC) was performed on formalin fixation and paraffin embedding (FFPE) samples to study the protein expression in the clinical specimens. Immunohistochemistry staining of KLK8 was performed using a primary antibody against KLK8 (1:100 dilution; ab150395; Abcam, Cambridge, UK) according to the manufacturer’s instructions. Briefly, FFPE pancreatic cancer tissues and paired adjacent noncancerous tissues were sectioned to a thickness of 4 μm. After routine deparaffinization, rehydration, antigen retrieval, and blocking with 3% hydrogen peroxide, the sections were then incubated with primary antibody which is specific for KLK8 (1:100 dilution; ab150395; Abcam, Cambridge, UK) overnight at 4°C and then with HRP-labeled secondary antibody. Finally, the sections were stained with diaminobenzidine and counterstained with hematoxylin. Staining was independently examined by two experienced investigators blinded to the clinical characteristics of the patients. The score for KLK8 staining was based on the integrated staining intensity and the percentage of positive cells. Staining intensity was scored as follows (16): 0 = no color; 1 = yellow; 2 = light brown; and 3 = dark brown. The proportion of immune-positive tumor cells (number of positively labeled tumor cells/number of total tumor cells) was scored as follows (17): 0, positive cells <5%; 1, 6%- 25% positive cells;2, 26%- 50% positive cells; 3, positive cells 51%- 75%; and 4>76%.). The comprehensive score was the product of staining intensity and average proportion of positive cells and expressed as follows (18): negative staining (0-2); weak expression (3-5); moderate expression (6-9); and strong expression (10-12).

### Cell Culture and Stable Transfection

The human pancreatic cancer cell line Mia-paca-2, Panc-1 and human embryonic kidney cell line 293T (293T) were obtained from Type Culture Collection of the Chinese Academy of Sciences (Shanghai, China). Mia-paca-2 and Panc-1 cells were cultured in DMEM (Invitrogen). All medium was supplemented with 10% fetal bovine serum (Gibco, USA) and 1% penicillin/streptomycin (Invitrogen). KLK8 overexpression was performed using a lentiviral packaging system. To construct overexpressing exogenous KLK8 cell lines, full-length KLK8 (NM_144505) was cloned into the expression vector Ubi-MCS-3FLAG-CBh-gcGFP-IRES-puromycin (Shanghai Genechem Co. Shanghai, China) and transfected into Mia-paca-2 and Panc-1 cell lines according to the manufacturer’s instructions. Briefly, Mia-paca-2 and Panc-1 cells were placed in 6-well plates at a density of 1 × 10^5cells/well the day before infection. The next day lentivirus were added in cell culture medium. Viruses were removed 24 h after infection and fresh cell culture medium was added. 72h after transfection, puromycin (2 µg/ml; Roche, USA) was added into the cell culture medium to generate stable KLK8-overexpression cell line four weeks later. Antibiotic-resistant cells were pooled for subsequent analysis.

### Western Blot Analysis

The cells were lysed, and proteins were extracted through standard protocols. The proteins were separated by SDS-polyacrylamide gel electrophoresis and subjected to western blot analyses. Protein bands were detected by the chemiluminescence method. Specific primary antibodies against KLK8(1:1000 dilution; ab150395; Abcam, Cambridge, UK),PI3K(1:1000 dilution; #4257; Cell Signaling Technology, Danvers, MA, USA), Akt (1:1000 dilution; ab8805; Abcam, Cambridge, UK), mTOR (1:1000 dilution; ab32028; Abcam, Cambridge, UK), p-PI3K(1:1000 dilution,P85 Tyr458; #17366; Cell Signaling Technology, Danvers, MA, USA), p-Akt(1:5000 dilution, Ser473; ab81283; Abcam, Cambridge, UK), p-mTOR (1:1000 dilution, Ser2448; ab109268; Abcam, Cambridge, UK), p-4EBP1(1:1000 dilution, Ser65; #9451; Cell Signaling Technology, Danvers, MA, USA), p-S6P-p70S6K (1:1000 dilution, Thr389; #9234; Cell Signaling Technology, Danvers, MA, USA), Notch1 (1:1000 dilution; ab52627; Abcam, Cambridge, UK), *c-myc* (1:1000 dilution; ab32072; Abcam, Cambridge, UK), cyclin D1 (1:1000 dilution; #2922; Cell Signaling Technology, Danvers, MA, USA), cleaved caspase-3 (1:500 dilution; #9664; Cell Signaling Technology, Danvers, MA, USA), cleaved caspase-9 (1:1000 dilution; #52873; Cell Signaling Technology, Danvers, MA, USA), Bax (1:1000 dilution; ab32503; Abcam, Cambridge, UK) were used. β-Actin (1:5000 dilution; sc-47778; Santa, Cruz, CA, USA) was used as a loading control. The chemiluminescent signals were detected with the chemiluminescence imaging system and quantified by Image J software (v1.37).

### Cell Counting Kit-8 (CCK8) Assay

The density of 1000 pretreated cells/well were seeded into a 96-well plate. The cells were incubated with CCK8 reagent (DOJINDO, Japan) at 37°C for 1 h and absorbance at 450 nm were measured using a microplate reader (BioTek, Vermont, USA) for the appropriate time.

### Colony Formation

Log phase Mia-paca-2 and Panc-1 cells were collected. The 500 cells were planted in each well of the 6-well plate and incubated at 37°C exposed to 5% CO_2_. After 14 days, the cells were fixed by 4% paraformaldehyde and stained by 0.1% crystal violet and the colonies were counted visually, with >100 cells/colony considered a clone.

### Apoptosis Assessment

Following transfected with KLK8 and vector plasmid, cells were washed with PBS 3 times and then stained using the Annexin V-FITC Apoptosis Detection Kit (BD Biosciences) according to the instruction. Then cells were analyzed with a FACS flow cytometer (BD Biosciences).

### Enzyme‐Linked Immunosorbent Assay

Mia-paca-2 and Panc-1 cells were plated in 12‐well plates and Enzyme‐linked immunosorbent assay (ELISA) was performed to detected the EGF levels in the supernatants after cultured for 48 hours using the Human EGF Quantikine ELISA Kit (#DEG00, R&D Systems, Minneapolis, MN, USA) according to manufacturer’s instructions

### Statistical Analysis

Data were expressed as means ± standard error of the mean (SEM) from at least three experiments. All statistical analyses were performed using SPSS 13.0 (SPSS Inc.). Independent samples t-test was used to compare control and treatment groups and one-way ANOVA was performed to compare the data of multiple groups. The Kaplan Meier estimation method was used for overall survival analysis, and a log-rank test was used to compare differences. P < 0.05 was considered to be statistically significant.

## Results

### KLK8 Was Associated With Pancreatic Cancer Progression and Patients’ Outcome

**Figure 1:**
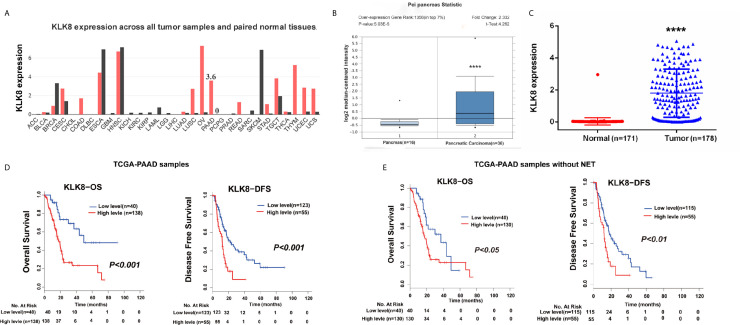
KLK8 was associated with PDAC tumorigenesis and prognosis in the TCGA-PAAD cohort. **(A)** KLK8 expression in different cancers and their paired normal tissues in the GEPIA. The height of bar represents the median expression of certain tumor type or normal tissue. **(B)** KLK8 expression of normal specimens and pancreatic carcinoma from an independent pancreatic dataset in the Oncomine database. The expression fold-change was 2.332. **(C)** In the TCGA-PAAD cohort, KLK8 was more significantly expressed in cancer patients (n=178) than in normal controls (n =171). **(D)** The TCGA-PAAD database has 178 pancreatic cancer samples, including 8 pancreatic neuroendocrine tumor (NET) samples. Overall survival (OS, left panel) and Disease-free survival (DFS, right panel) were compared between patients with low and high KLK8 expression in the TCGA-PAAD cohort (n=178). **(E)** We deleted these 8 NETs, and the remaining cohort consisted of 170 primary PDACs samples (TCGA-PAAD cohort without NETs, n=170) were used for performing the survival analysis. OS (left panel) and DFS (right panel) was compared between patients with low and high KLK8 expression. ****p < 0.0001 *vs* normal.

Based on the indicated role of KLK8 in malignant disease found in GEPIA (http://gepia.cancer-pku.cn/detail.php?gene=KLK8) (**Figure 1A**), we analyzed the expression of KLK8 in the independent public dataset from Oncomine (https://www.oncomine.org/resource/main.html) and found that KLK8 expression was elevated in the pancreatic cancer tissue samples in comparison to the normal pancreas (**Figure 1B**, P<0.0001). To further examine the potential relationship between KLK8 and PAAD, we analyzed data from the TCGA-PAAD cohort which was replenished by GTEX database, and found that KLK8 was significantly upregulated in tumor tissues compared to normal tissues (**Figure 1C**, P<0.0001). Then we evaluated the relationship between KLK8 expression and patients’ outcomes. The Kaplan–Meier curve analysis of the TCGA-PAAD database indicated that higher KLK8 expression in PAAD was correlated with shorter OS and DFS rates (P<0.01, **Figure 1D**). Notably, there are 8 pancreatic neuroendocrine tumor (NET) samples in the TCGA-PAAD database, which exhibit low KLK8 expression. NET is known to have a very different prognosis as compared to PDAC (19). Therefore, we deleted these 8 NETs, and the remaining cohort consisted of 170 primary PDACs samples were used for performing the survival analysis. As shown in **Figure 1E**, we found that higher KLK8 expression in PDAC was correlated with shorter OS (P < 0.05) and DFS (P < 0.01) rates. These results suggest that KLK8 is highly expressed in pancreatic cancer and is correlated with the prognosis of patients with pancreatic cancer.

### KLK8 Was Elevated in Pancreatic Cancer Tissues at Both the mRNA and Protein Levels

**Figure 2:**
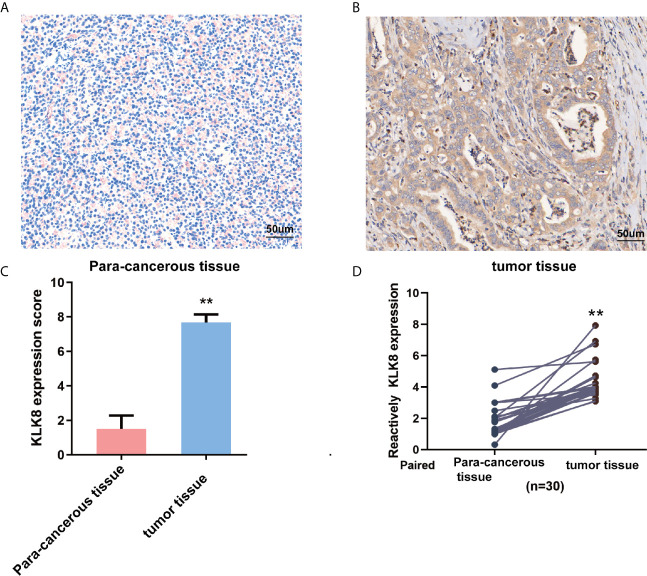
KLK8 expression in pancreatic cancer tissues and para-cancerous pancreas. **(A, B)** Representative immunohistochemistry staining of KLK8 in pancreatic cancer tissues and para-cancerous pancreas (n=20). **(A)** showed negative KLK8 staining in para-cancerous pancreas. **(B)** showed strong positive KLK8 staining in PDAC tissues. **(C)** Quantified data of the score for KLK8 staining. **(D)** Quantitative real-time PCR detection of KLK8 mRNA expression in paired human PDAC tissue samples and para-cancerous pancreas (n=30). Data were presented as the mean ± SEM. **p < 0.01 *vs* Non-tumor tissue.

To further investigate the expression of KLK8 in pancreatic cancers, KLK8 protein expression was assessed in 20 pancreatic cancer tissues and para-cancerous pancreas by IHC staining. Compared with normal tissues, the level of KLK8 were significantly increased in pancreatic cancer tissues (**Figures 2A–C**, P<0.01). We also performed H&E staining of sequential sections of those used for IHC staining. As shown in **Supplemental Figures S1A, B**, it was found that the tumor tissues exhibited typical pancreatic ductal adenocarcinoma. There were full of exocrine portions, plenty of infiltrating lymphocytes and some epithelioid cells in the para-cancerous tissues. Then, KLK8 mRNA expression was determined in 30 paired PDAC tissues and matched para-cancerous pancreas. It was found that KLK8 mRNA levels were significantly increased in pancreatic cancer tissue samples as comparison to the adjacent non-tumor tissues (**Figure 2D**, p < 0.01). These findings were consistent with the data obtained from the public datasets (**Figure 1**).

### KLK8 Exerted Pro-Proliferation and Anti-Apoptotic Functions in Pancreatic Cancer Cells

**Figure 3:**
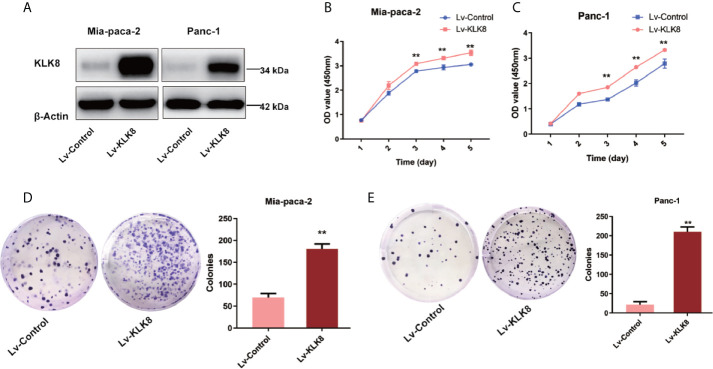
KLK8 overexpression promoted the proliferation of PDAC cells. KLK8 overexpression was induced with recombinant Lentivirus infection (Lv-KLK8) in human pancreatic cancer cell lines Mia-paca-2 and Panc-1, and an empty adenovirus served as control (Lv-Control). **(A)** Mia-paca-2 and Panc-1 cells transfected with KLK8 overexpression vectors were validated at protein level by western blot analysis. **(B, C)** Cell proliferation was detected by CCK8 Assay in Mia-paca-2 **(B)** and Panc-1 **(C)** cells. **(D, E)** Colony formation was detected in Mia-paca-2 **(D)** and Panc-1 **(E)** cells. Data were presented as the mean ± SEM (n=3). **p < 0.01 *vs* Lv-control.

Abnormal cell proliferation and apoptosis are characteristics of human malignant tumor (20). We then determined whether elevated KLK8 expression could influence the proliferation and apoptosis of pancreatic cancer cells by using KLK8-overexpressed Mia-paca-2 and Panc-1 cell lines. The efficacy of KLK8 overexpression in two cell lines was confirmed by western blot analysis (**Figure 3A**). We performed CCK-8 and colony formation assay to assess the effects of KLK8 in pancreatic cell proliferation. As shown in **Figures 3B, C**, a significant promotion of cell proliferation was observed in the KLK8-overexpression group in comparison to the control group. In addition, the number of cell colonies were significantly increased in both Mia-paca-2 and Panc-1 pancreatic cancer cells overexpressed with KLK8 (**Figures 3D, E**).

**Figure 4:**
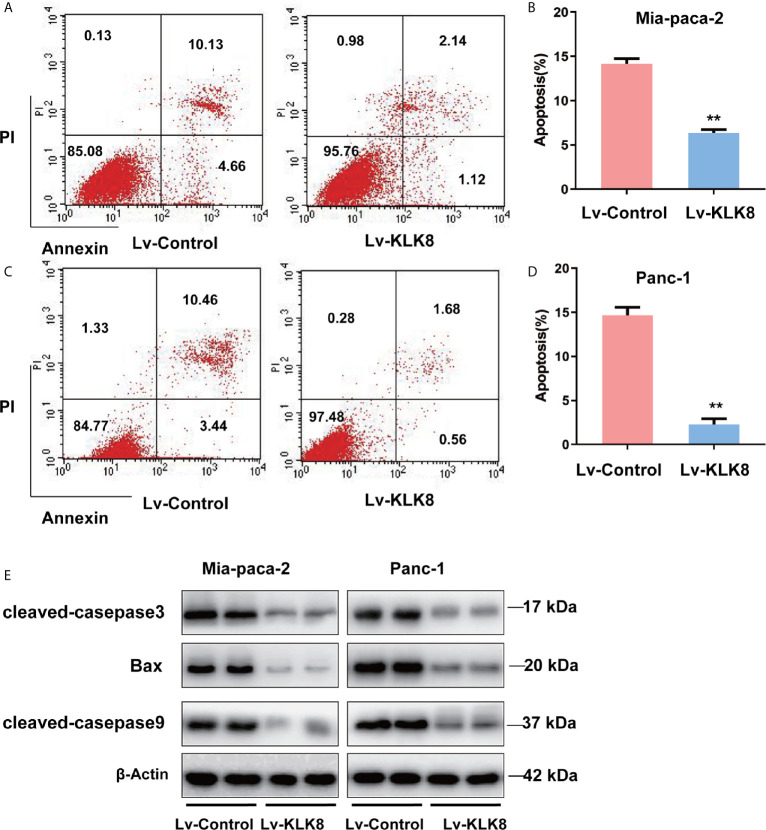
KLK8 overexpression inhibited apoptosis of PDAC cells. KLK8 overexpression was induced with recombinant Lentivirus infection (Lv-KLK8) in human pancreatic cancer cell lines Mia-paca-2 **(A, B)** and Panc-1 **(C, D)**, and an empty adenovirus served as control (Lv-Control). Cell apoptosis was determined by Annexin V-FITC and PI double staining analysis performed by flow cytometry. Representative flow cytometry images were shown **(A, C)**. **(B, D)** demonstrated the quantified data of cell apoptosis. **(E)** pro-apoptotic markers cleaved caspase-3, cleaved caspase-9 and Bax assessed by western blot. Data are expressed as the mean ± SEM (n=3). **p < 0.01 *vs* Lv-control.

We then clarified the effect of KLK8 overexpression on pancreatic cancer apoptosis. As shown in **Figures 4A–D**, compared with control group, the percentage of apoptosis cells was significantly reduced in KLK8 overexpressed pancreatic cancer cells. In addition, western blot assay showed that compared with control group, pro-apoptotic markers cleaved caspase-3, cleaved caspase-9 and Bax significantly declined in KLK8-overexpression Mia-paca-2 and Panc-1 cells (**Figure 4E**).

### KLK8 Accelerated Cell Growth and Inhibited Apoptosis *via* PI3K-Akt-mTOR Signaling Pathway in Pancreatic Cancer Cells

**Figure 5:**
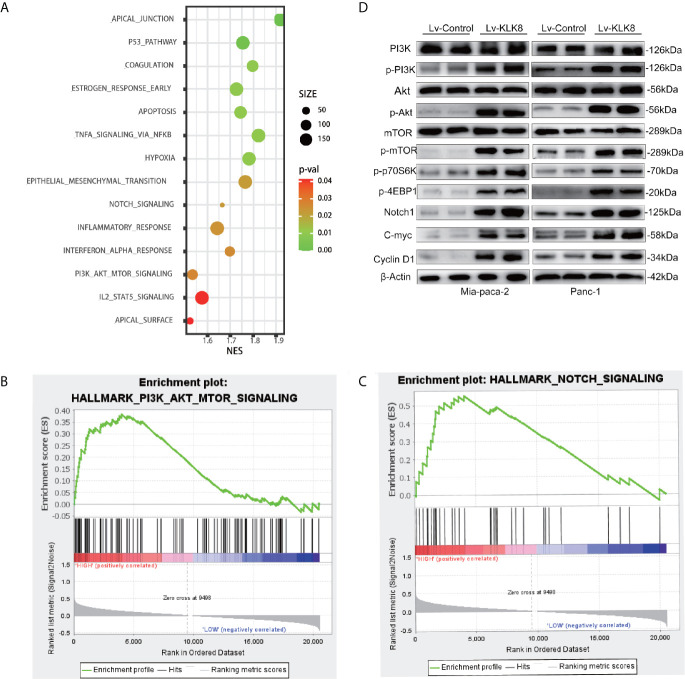
Significantly-altered pathways were predicted in PDAC and verified in KLK8-overexpressed pancreatic cancer cells. **(A)** Gene sets enriched in the transcriptional profiles of tumors belonging to the top KLK8 high-expression group, compared with the bottom-expression group in the TCGA dataset. Shown are the NES (normalized enrichment score) values for each pathway using the Hallmark gene sets. The functional annotations of KLK8 positive and negative expression in PDAC was predicted. A nominal p value of <0.05 is considered statistically significant. **(B, C)** GSEA highlighted positive association of increased KLK8 expression levels with PI3K-Akt-mTOR **(B)** and Notch **(C)** signal pathways. **(D)** levels of key proteins in PI3K-AKT-mTOR and Notch signaling pathways were examined in KLK8-overexpressed Mia-paca-2 and Panc-1 cells using western blot. NES = normalized enrichment score. (n=3).

To gain an insight into the mechanisms by which KLK8 promoted PDAC progression, the gene expression in PDAC tissues with high expression of KLK8 and those with low expression of KLK8 was analyzed by gene set enrichment analysis (GSEA) based on the TCGA database. GSEA results showed that 14 enriched pathways were differentially expressed according to diverse KLK8 expression levels (**Figure 5A**, p<0.05). Notably, KLK8 was positively associated with PI3K-AKT-mTOR and Notch signaling pathways, both of which are known to play critical roles in cell proliferation and apoptosis (**Figures 5B, C**). We observed significantly enhanced phosphorylated PI3K, phosphorylated Akt and phosphorylated mTOR expression in Mia-paca-2 and Panc-1 cells overexpressed with KLK8. KLK8 overexpression also led to significant increases in phosphorylated 4EBP1 and phosphorylated S6P-p70S6K, two of the most distinctive downstream targets of mTOR, in pancreatic cancer cell lines (**Figure 5D**). In addition, KLK8 overexpression also led to significant increases in Notch-1 protein expression. C-myc and Cyclin D1, two downstream targets of Notch signaling, were increased in pancreatic cancer cells overexpressed with KLK8 (**Figure 5D**). These findings suggest that KLK8 overexpression activates PI3K-Akt-mTOR and Notch pathways (**Figure 5D**).

**Figure 6:**
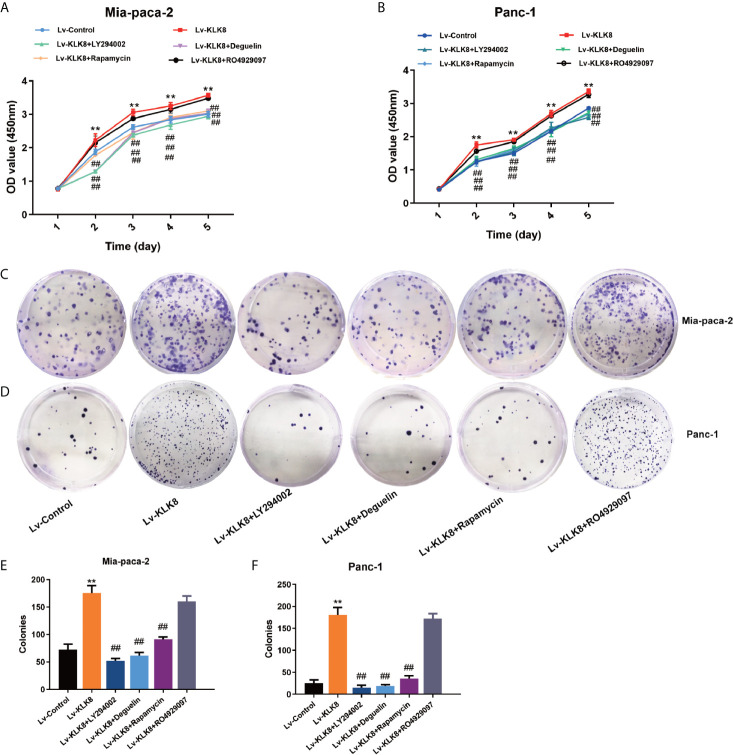
KLK8 promoted pancreatic cancer cells proliferation *via the* activation of the PI3K-Akt-mTOR pathway. Mia-paca-2 and Panc-1 cells in Lv-KLK8 group were treated with PI3K inhibitor LY294002(75 μM), Akt inhibitor Deguelin (500 nM), mTOR inhibitor Rapamycin (100 nM) or Notch inhibitor RO4929097 (10 μM) in Mia-paca-2 and Panc-1 cells. **(A, B)** Cell proliferation was detected by CCK8 Assay in Mia-paca-2 **(A)** and Panc-1 **(B)** cells. **(C, D)** Colony formation was detected in Mia-paca-2 **(C)** and Panc-1 **(D)** cells. **(E, F)** demonstrated the quantified data of cell colonies. Data were presented as the mean ± SEM (n=3). **p < 0.01 *vs* Lv-control; p < 0.05, ^##^p < 0.01 *vs* Lv-KLK8.

Next, we explored whether activation of PI3K-Akt-mTOR and Notch signaling pathways contributed to the pro-proliferation and anti-apoptotic functions of KLK8 in pancreatic cancer cells. Both CCK8 assay and colony formation assay showed that the pro-proliferation effects of KLK8 on pancreatic cells were counteracted by PI3K inhibitor LY294002 (75 μM), Akt inhibitor Deguelin (500 nM), and mTOR inhibitor Rapamycin (100 nM) (**Figure 6**). However, Notch inhibitor RO4929097 (10 μM) had no significant effect on KLK8-induced pro-proliferation in pancreatic cells (**Figure 6**). We also measured the effects of the specific inhibitors on cell proliferation in Lv-control treated cells. As shown in **Supplemental Figures S2A, B**, it was found that only PI3K inhibitor LY294002 at the dose of 75 μM significantly inhibited cell proliferation in both Mia-paca-2 and Panc-1 cells treated with Lv-control. However, Akt inhibitor Deguelin (500 nM), mTOR inhibitor Rapamycin (100 nM), and Notch inhibitor RO4929097 (10 μM) had no significant effect on cell proliferation in Lv-control treated pancreatic cancer cells.

**Figure 7:**
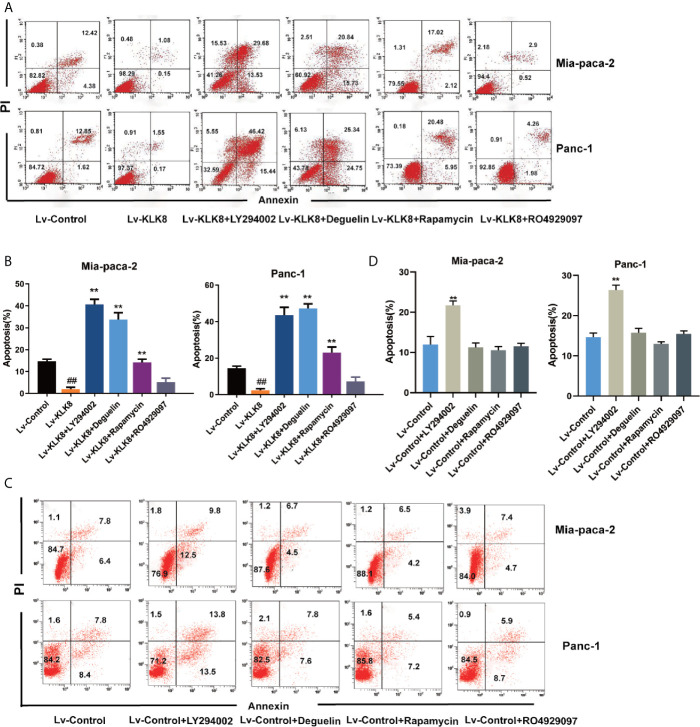
KLK8 suppressed pancreatic cancer cells apoptosis through the activation of PI3K-Akt-mTOR pathway. Mia-paca-2 and Panc-1 cells in Lv-Control and Lv-KLK8 were treated with PI3K inhibitor LY294002 (75 μM), Akt inhibitor Deguelin(500 nM), mTOR inhibitor Rapamycin (100 nM) or Notch inhibitor RO4929097 (10 μM). Cell apoptosis was determined by Annexin V-FITC and PI double staining analysis performed by flow cytometry. Representative flow cytometry images were shown **(A, C)**. **(B, D)** demonstrated the quantified data of cell apoptosis. Data are presented as means ± SEM (n = 3). **p < 0.01 *vs* Lv-control; p < 0.05, ^##^p < 0.01 *vs* Lv-KLK8.

**Figure 8:**
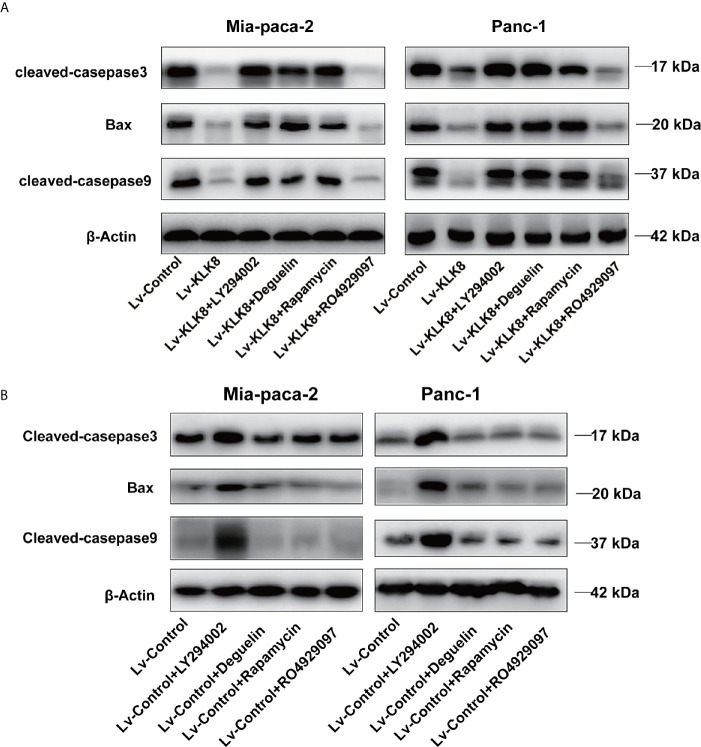
KLK8 decreased protein levels of pro-apoptotic proteins through the activation of PI3K-Akt-mTOR pathway. Mia-paca-2 and Panc-1 cells in Lv-KLK8 **(A)** and Lv-Control **(B)** were treated with PI3K inhibitor LY294002(75 μM), Akt inhibitor Deguelin (500 nM), mTOR inhibitor Rapamycin (100 nM) or Notch inhibitor RO4929097 (10 μM). **(A, B)** protein levels of pro-apoptotic markers cleaved caspase-3, cleaved caspase-9 and Bax were assessed by western blot.

We then examined the effects of the specific inhibitors on cell apoptosis. As shown in **Figures 7**, **8**, PI3K inhibitor LY294002 at the dose of 75 μM significantly promoted cell apoptosis in both Mia-paca-2 and Panc-1 cells treated with Lv-control. In Lv-KLK8 treated pancreatic cancer cells, we found that LY294002 reversed the anti-apoptotic effect of KLK8 overexpression. Moreover, KLK8-overexpressed pancreatic cancer cells treated by LY294002 showed higher levels of apoptosis than Lv-control treated cells (**Figures 7A, B**, **8A**).

Both Deguelin (500 nM) and Rapamycin (100 nM) had no significant effect on cell apoptosis in Lv-control treated pancreatic cancer cells (**Figures 7C, D**, **8B**). In Lv-KLK8 treated pancreatic cancer cells, we found that Deguelin and Rapamycin reversed the anti-apoptotic effect of KLK8 overexpression (**Figures 7A, B**, **8A**). Moreover, KLK8-overexpressed pancreatic cancer cells treated by Deguelin showed higher levels of apoptosis than Lv-control treated Mia-paca-2 and Panc-1 cells (**Figures 7A, B**). KLK8-overexpressed Panc-1 cells treated by Rapamycin also showed higher levels of apoptosis than Lv-control treated cells (**Figures 7A, B**).

However, Notch inhibitor RO4929097 (10 μM) had no significant effect on cell apoptosis in Lv-control treated pancreatic cancer cells (**Figures 7C, D**). In addition, RO4929097 had no significant effect on KLK8-induced anti-apoptotic function in pancreatic cells (**Figures 7A, B**, **8A**).

### EGF Signaling Contributes to KLK8-Induced Activation of PI3K-Akt-mTOR Signaling Pathway and KLK8-Induced Pro-Proliferation and Anti-Apoptotic Effects in Pancreatic Cancer Cells

**Figure 9:**
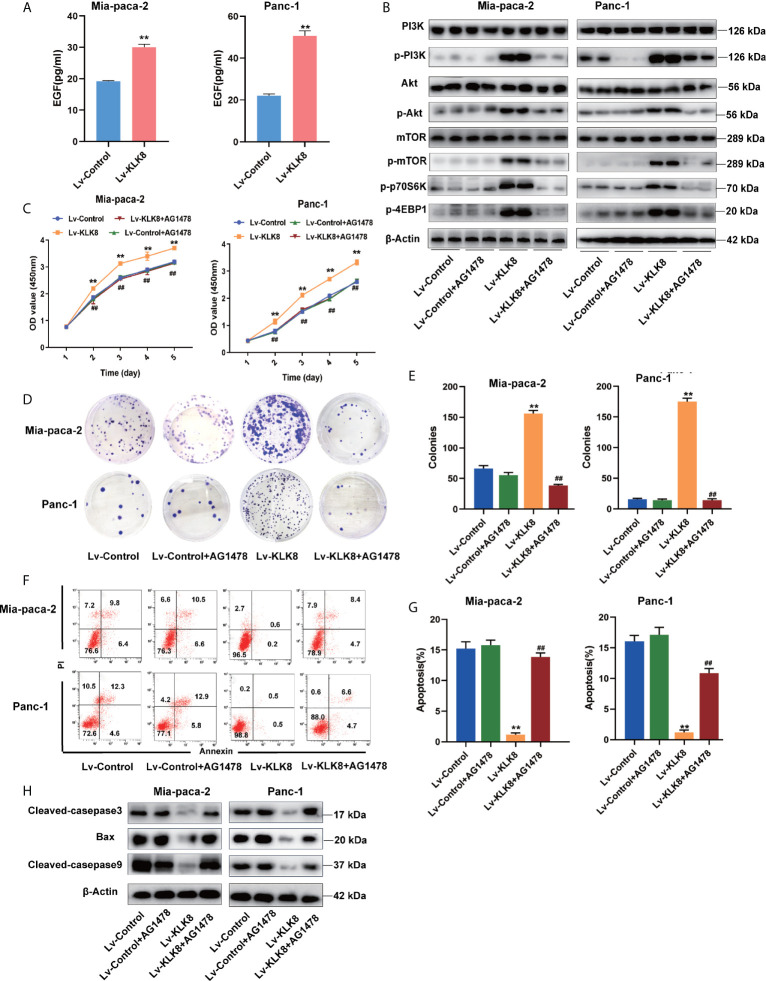
EGF signaling contributes to KLK8-induced activation of PI3K-Akt-mTOR signaling pathway and KLK8-induced pro-proliferation and anti-apoptotic effects in pancreatic cancer cells. Mia-paca-2 and Panc-1 cells were treated with EGFR antagonist AG1478 (100 nM). Twenty-four hours later, cells were harvested for measuring cell protein level, proliferation and apoptosis. **(A)** Expression level of EGF in Mia-paca-2 and Panc-1 cells were evaluated by ELISA assay. **(B)** Levels of key proteins in PI3K-AKT-mTOR signaling pathways were examined in Mia-paca-2 and Panc-1 cells using western blot **(C)** Cell proliferation was detected by CCK8 assay in Mia-paca-2 and Panc-1 cells. **(D)** Colony formation was detected in Mia-paca-2 and Panc-1 cells. **(E)** Demonstrated the quantified data of cell colonies. **(F)** Cell apoptosis was determined by Annexin V-FITC and PI double staining analysis performed by flow cytometry. Representative flow cytometry images were shown. **(G)** Demonstrated the quantified data of cell apoptosis. **(H)** Pro-apoptotic markers cleaved caspase-3, cleaved caspase-9 and Bax assessed by western blot. Data were presented as the mean ± SEM (n=3). **p <0.01 *vs* Lv-control; p < 0.05, ^##^p < 0.01 vs Lv-KLK8.

**Figure 10:**
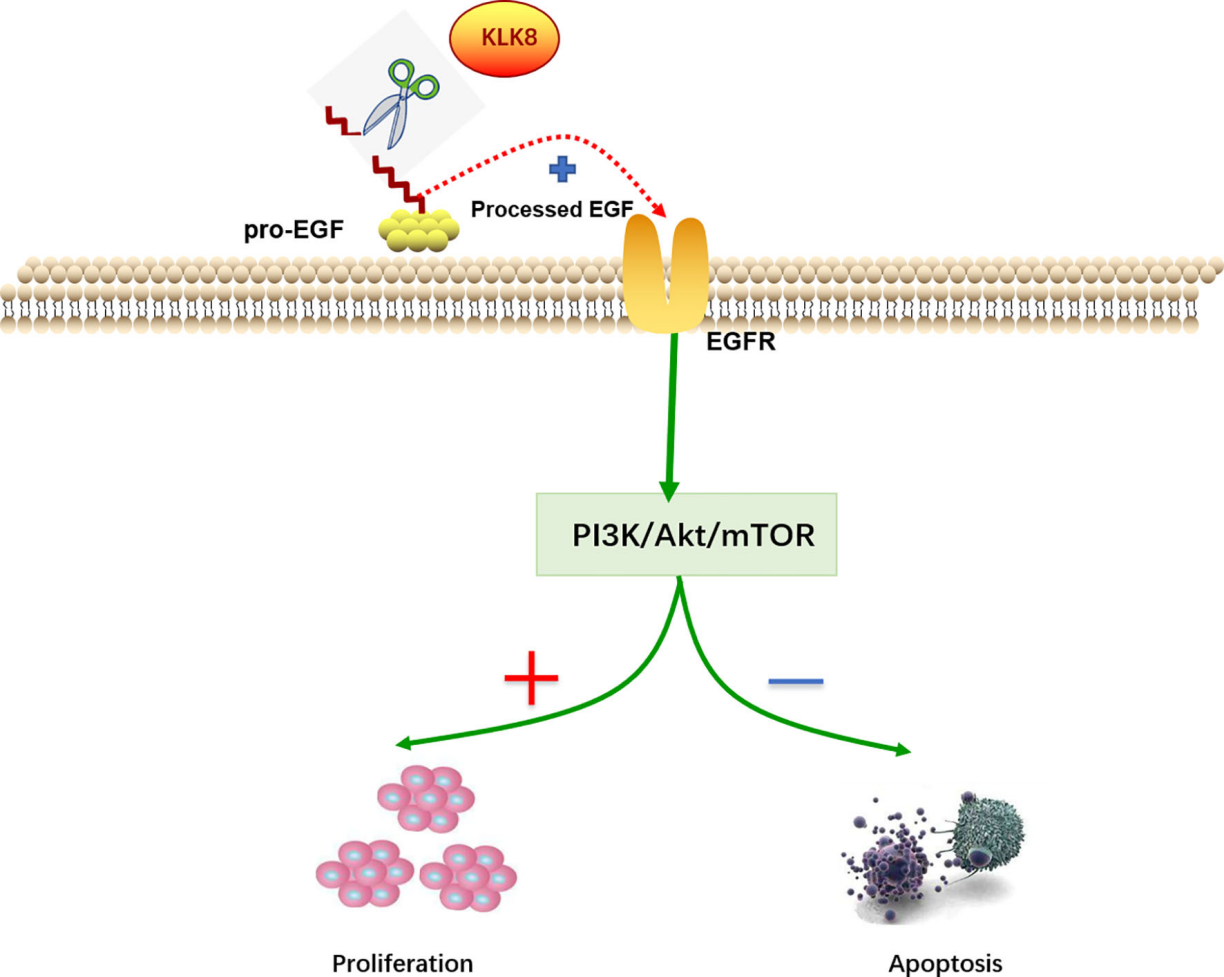
Molecular mechanisms of KLK8-induced pro-proliferation and anti-apoptotic effects in pancreatic cancer cells. KLK8-mediated release of mature EGF contributes to the activation of PI3K-Akt-mTOR signaling pathway, thereby promoting the proliferation and inhibiting the apoptosis of pancreatic cancer cells.

As a secreted serine protease, KLK8 is known to mediate the proteolytic process of pro-EGF into mature EGF (21).EGF is known to activate PI3K/Akt/mTOR pathway in a variety of cancers (22–25). Thus, we further investigated whether EGF signaling pathway contributes to KLK8-induced proliferation and anti-apoptotic effects in pancreatic cancer cells. As shown in **Figure 9A**, it was found that overexpression of KLK8 in Mia-paca-2 and Panc-1 cells significantly increased EGF levels in the culture media. Western blot results demonstrated that KLK8-induced activation of PI3K/Akt/mTOR pathway was profoundly blocked by AG1478, the specific EGF receptor (EGFR) antagonist (**Figure 9B**). In addition, it was found that EGFR antagonist AG1478 significantly attenuated the pro-proliferation and anti-apoptotic effects of KLK8 overexpression in both Mia-paca-2 and Panc-1 cells (**Figures 9C–H**). These findings indicate that EGF signaling contributes to KLK8-induced activation of PI3K-Akt-mTOR signaling pathway and KLK8-induced pro-proliferation and anti-apoptotic effects in pancreatic cancer cells (**Figure 10**).

## Discussion

Pancreatic cancer continues to have a poor 5-year survival rate despite its rising incidence (26, 27). By 2030, it is estimated to become the second leading cause of cancer related deaths (5). Pancreatic resection is still the only curative intent therapy for PDAC patients. However, pancreatic resection is complex and carries with it the risk of major morbidity and mortality (28). Thus, there is a desperate need for investigating the pathogenesis and identifying molecular biomarkers of PDAC to facilitate early diagnosis, prognosis prediction, and the development of effective therapeutic strategies for PDAC patients.

KLK8, also known as neuropsin, is a member of human kallikrein-related peptidase (KLKs) family which has been related to malignant behavior at multiple stages of tumor progression, including proliferation, migration and angiogenesis (29, 30). Previous studies have found that abnormal expression of KLK8 was associated with several malignancies, including ovarian, cervical, gland and lung cancers (12, 31–34).However, the expression level and prognostic significance of KLK8 in PDAC are still unknown. In this study, we identified up-regulated KLK8 expression in pancreatic cancer compared with adjacent tissues through TCGA database, which was further confirmed by using clinical samples. Furthermore, we found that high KLK8 expression predicts poorer OS and DFS in pancreatic cancer patients. These results indicated that KLK8 could be a prognostic marker for PDAC. Similar to our findings, several studies have confirmed that the upregulation of KLK8 was related to poorer cancer prognosis. For example, KLK8 has been recognized as a poor prognostic marker for lung and breast cancer (15, 31). But in other tumors, such as ovarian cancer, the elevated expression of KLK8 is a favorable prognostic marker (35). These results suggest that KLK8 may play different roles in different cancers, and the aberrant expression of KLK8 may serve as a potential clinical biomarker for cancer diagnosis or prognosis.

KLK family members have been implicated in the pathogenesis and progression of malignant tumor (36, 37). For example, overexpression of KLK7 is found to stimulate colon cancer cell proliferation both *in vivo* and *in vitro* (38). KLK13 enhances the invasiveness and motility of lung cancer *via* increasing laminin degradation and N-cadherin expression (39). KLK5 promotes metastatic dissemination of Oral squamous cell carcinoma(OSCC) by promoting loss of junctional integrity through cleavage of desmoglein 1 (40). KLK14 acts at the cleavage site of PAR-2 to induce ERK1/2 activation, thus promoting colon cancer proliferation (41). As for KLK8, it can facilitate colorectal cancer (CRC) cell proliferation, migration and invasion *in vitro* (9). In this study, by using two pancreatic cancer cell lines, we demonstrated for the first time that overexpression of KLK8 significantly inhibited PDAC cell apoptosis, meanwhile profoundly promoted PDAC cell proliferation. These data suggest that KLK8 may promote tumor growth and suppress tumor apoptosis, and may be a potential molecular target in therapy for pancreatic cancer.

Notably, our recent study has shown that KLK8 overexpression in coronary artery endothelial cells cleaves VE-cadherin, thus leading to endothelial cell damage and endothelial hyperpermeability (42). In contrast, the present study showed that KLK8 overexpression significantly increased cell viability of pancreatic cancer cells. Previous studies also demonstrate that KLK8 overexpression significantly increases cell viabilities in neonatal cardiomyocytes and cardiac fibroblasts (21, 42). Taken together, these findings suggest that KLK8 might modulate cell functions in a cell type-dependent manner.

The activity of KLK family members is typically controlled by itself or other proteases in the proteolytic activation cascade (43, 44). For example, KLK5 is thought to initiate the cascade reaction through auto-activation, and is activated by other proteases including the transmembrane serine protease matriptase and matrix metalloproteases (MMPs) (45). KLK5 activates other KLKs, such as KLK7 and KLK14 (46).Pro-KLK8 has been found to be activated by KLK5 and other proteases such as lysyl endopeptidase and MMP9 (47). The present study found that lentivirus-mediated KLK8 overexpression exhibited significant pro-proliferation and anti-apoptotic effects in pancreatic cancer cells, suggesting that the active form of KLK8 is increased after KLK8 overexpression. Whether KLK8 is activated through auto-activation or by other proteases merits further investigation.

Phosphatidylinositide 3 kinases (PI3Ks) and their downstream mediators Akt and mammalian target of rapamycin (mTOR) are well-known to regulate cell proliferation, apoptosis, homeostasis and metabolism (48). Previous studies have demonstrated that activation of PI3K/AKT/mTOR signaling pathway facilitates pancreatic cancer cell proliferation. In contrast, blockade of PI3K/AKT/mTOR signaling pathway promotes pancreatic cancer cell death (49–51). Overexpression of KLK8 has been found to induce Akt activation under Hypoxia/Reoxygenation (H/R) stimulation in neonatal rat cardiomyocytes (8). In the present study, GSEA analysis and western blot assay revealed that KLK8 overexpression resulted in the activation of PI3K/AKT/mTOR signaling pathway in pancreatic cancer cells. In addition, the pro-proliferation and anti-apoptotic functions of KLK8 were reversed by inhibitors targeting PI3K, Akt and mTOR. These findings suggest that elevated KLK8 may exert the pro-proliferation and anti-apoptotic effects in pancreatic cancer cells through activating PI3K-Akt-mTOR signaling pathway.

In addition, we noticed that PI3K inhibitor LY294002 at the dose of 75 μM significantly promoted cell apoptosis in both Mia-paca-2 and Panc-1 cells treated with Lv-control. KLK8-overexpressed pancreatic cancer cells treated by LY294002 showed higher levels of apoptosis than Lv-control treated cells. These findings suggest that PI3K inhibitor LY294002 may promote apoptosis independently from the signaling mediated by KLK8. As for Akt inhibitor Deguelin and mTOR inhibitor Rapamycin, both inhibitors had no significant effect on cell apoptosis in Lv-control treated pancreatic cancer cells. However, KLK8-overexpressed pancreatic cancer cells treated by Deguelin showed higher levels of apoptosis than Lv-control treated Mia-paca-2 and Panc-1 cells. KLK8-overexpressed Panc-1 cells treated by Rapamycin also showed higher levels of apoptosis than Lv-control treated cells. These findings suggest that Deguelin and Rapamycin may cause KLK8 to functionally switch from being anti-apoptotic to pro-apoptotic in pancreatic cancer cells, a possibility that merits further investigation.

As a secreted serine protease, KLK8 is known to cleave several membrane proteins including protease-activated receptors (PARs), neuregulin-1, synaptic adhesion molecule L1, Ephrin type-B receptor 2, and VE-cadherin (10, 21, 42, 44). In addition, the proteolytic process of pro-EGF into mature EGF can also be mediated by KLK8. EGF/EGFR signaling pathway has been known to promote the proliferation of pancreatic cancers *via* activating PI3K/AKT pathway. In this study, we found that EGF levels significantly increased in KLK8-overexpression Mia-paca-2 and Panc-1 cells. Inhibition of EGFR could block KLK8-induced activation of PI3K/Akt/mTOR pathway and attenuated the pro-proliferation and anti-apoptotic effects of KLK8 overexpression in both Mia-paca-2 and Panc-1 cells. Taken together, these results indicate that the effects of KLK8 on PI3K/Akt/mTOR activation, pancreatic cell proliferation and apoptosis might be, at least partly, due to KLK8-mediated release of mature EGF.

Notch signaling pathway also plays an important role in the occurrence and progression of pancreatic cancer (52–54). In the present study, GSEA analysis and western blot assay revealed that KLK8 overexpression resulted in the activation of Notch signaling pathway. However, Notch inhibitor didn’t influence the KLK8-induced effects in pancreatic cancer cells. These results suggest that the pro-proliferation and anti-apoptotic functions of KLK8 may not be dependent on activation of Notch signaling pathway. Notably, our GSEA analysis data showed that KLK8 overexpression might also lead to the activation of EMT (epithelial-mesenchymal transition), glycolysis and KRAS signaling pathway, which have been implicated in the pathogenesis and progression of pancreatic cancers (17, 55–57). Whether these processes and the related signaling pathways contribute to the KLK8-induced pro-proliferation and anti-apoptotic effects in pancreatic cancers merits further investigation.

## Conclusions

In summary, our findings indicate that KLK8 overexpression exerts pro-proliferation and anti-apoptotic functions in pancreatic cancer cells *via* EGF signaling-dependent activation of PI3K/Akt/mTOR pathway. Positive KLK8 staining is associated with PDAC progression and predict poorer survival in patients, thus providing additional evidence for patient-tailored therapeutic strategies.

## Data Availability Statement

The original contributions presented in the study are included in the article/**Supplementary Material**. Further inquiries can be directed to the corresponding authors.

## Ethics Statement

This study was approved by the Ethics Committee of Fudan University Shanghai Cancer Center. The patients/participants provided their written informed consent to participate in this study. Written informed consent was obtained from the individual(s) for the publication of any potentially identifiable images or data included in this article.

## Author Contributions

QH was responsible for conducting the study, under the supervision of XZ and PX and contributed to the experimental design. QH, TL and YL did the experiments and analyzed the data. QH and XS wrote the paper. All authors contributed to the article and approved the submitted version.

## Funding

This work was supported by grants from National Natural Science Foundation of China (No. 81471852, No.31671213, No.31871156) and Shanghai Natural Science Foundation Program (20ZR1412900).

## Conflict of Interest

The authors declare that the research was conducted in the absence of any commercial or financial relationships that could be construed as a potential conflict of interest.

## Publisher’s Note

All claims expressed in this article are solely those of the authors and do not necessarily represent those of their affiliated organizations, or those of the publisher, the editors and the reviewers. Any product that may be evaluated in this article, or claim that may be made by its manufacturer, is not guaranteed or endorsed by the publisher.

## Abbreviations

PDAC, Pancreatic ductaladenocarcinoma; KLK8, Kallikrein-related peptidase 8; GSEA, gene set enrichment analysis; PI3K, Phosphatidylinositide 3 kinases; mTOR, mammalian target of rapamycin; KLKs, kallikrein-related peptidases; PAAD, Pancreatic Cancer; OSCC, Oral squamous cell carcinoma; CRC, Colorectal cancer; H/R, Hypoxia/Reoxygenation; EMT, epithelial-mesenchymal transition; EGF, epidermal growth factor; EGFR, epidermal growth factor Receptor.
