# Supplementary figures and images for: Upregulation of KLK8 Predicts Poor Prognosis in Pancreatic Cancer
Source: Front Oncol. 2021 Jul 30;11:624837. doi: 10.3389/fonc.2021.624837 (PMC8362328; doi:10.3389/fonc.2021.624837)

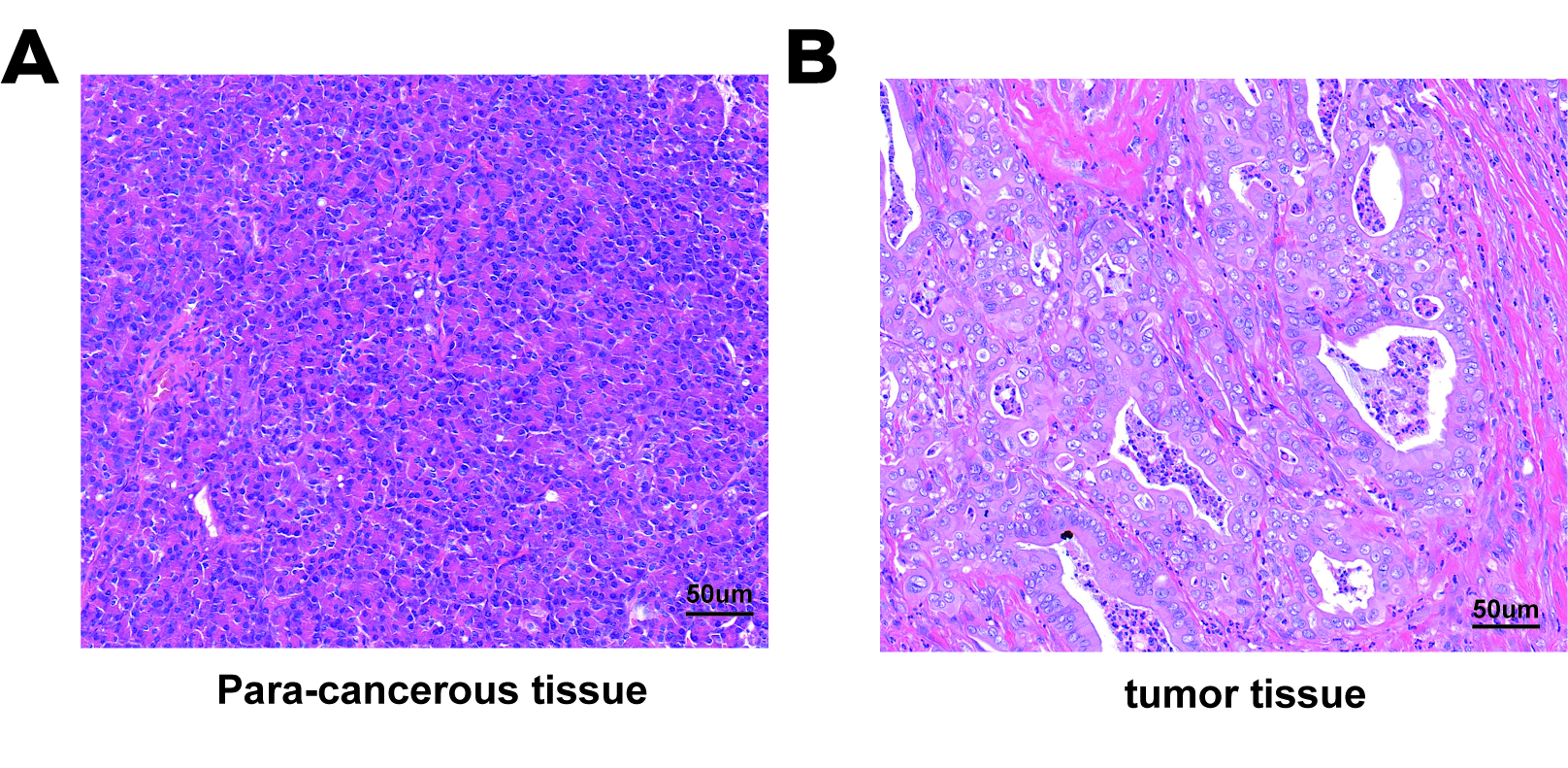

Supplement: Supplementary file 1 [file Image_1.tif]

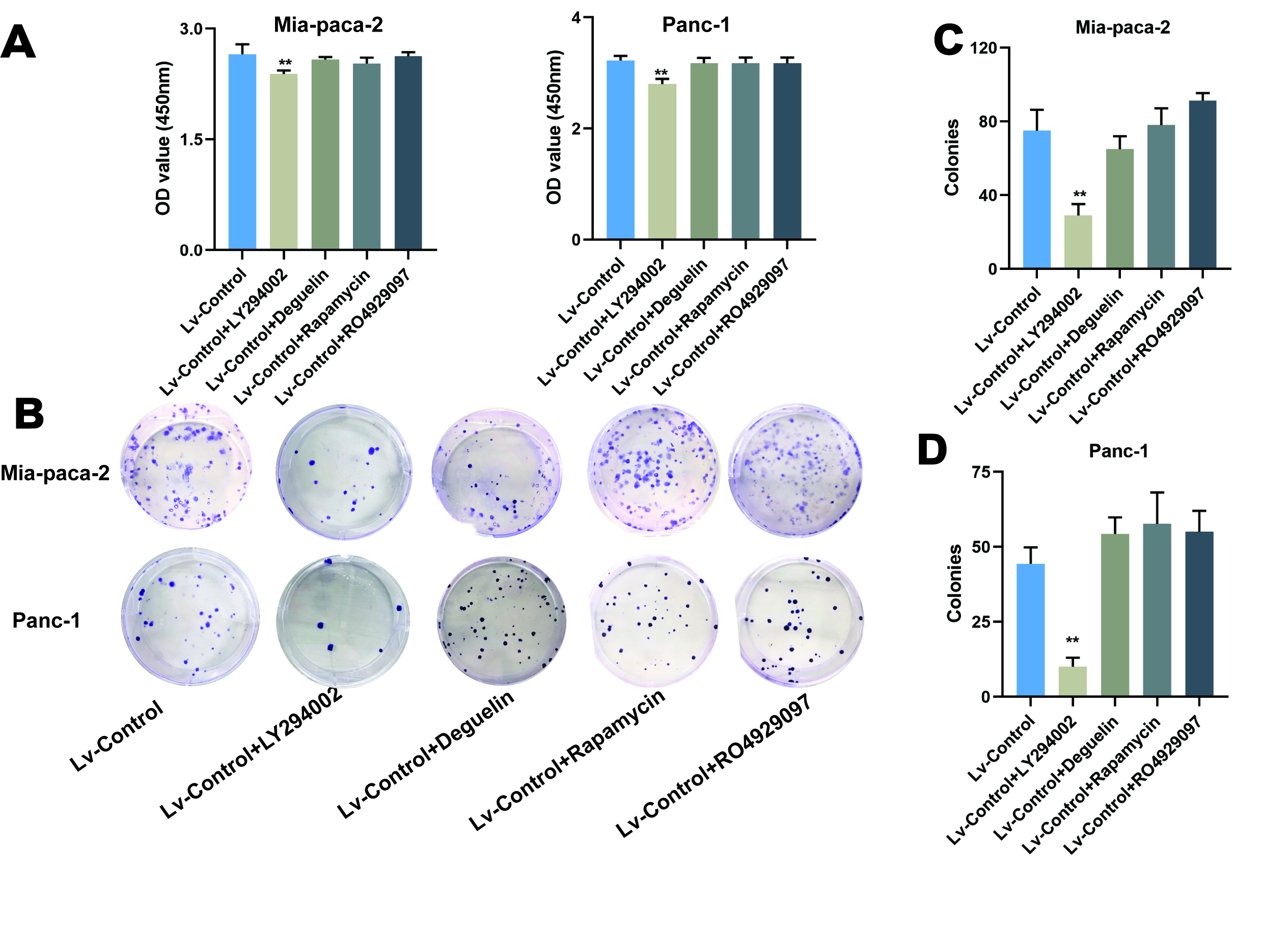

Supplement: Supplementary file 2 [file Image_2.tif]
